# Flavonoid production via solid-state fermentation of agro-industrial waste with biopesticidal fungi: screening, optimization and scale-up

**DOI:** 10.1007/s00449-026-03316-8

**Published:** 2026-03-23

**Authors:** Fabíola Ribeiro de Oliveira, Arnau Sala, Clarissa Okino-Delgado, Teresa Gea, Fernanda Casciatori

**Affiliations:** 1 Graduate Program of Chemical Engineering, Federal University of São Carlos (UFSCar), Rod. Washington Luiz km 235 SP 310, Bairro Monjolinho, São Carlos, SP 13565-905 Brazil; 2 GICOM - Research Group on Bioconversion of Organic Residues and Advanced Materials, Department of Chemical, Biological and Environmental Engineering, Universitat Autònoma de Barcelona, Edifici Q, Campus de Bellaterra, 08193 Cerdanyola del Vallès, Spain

**Keywords:** biodefensives, solid-state fermentation, process optimization, phenolic compounds

## Abstract

Flavonoids are bioactive compounds with significant applications in agriculture due to their antimicrobial properties, ability to mitigate abiotic stress and role in promoting plant-microbe interactions. This study explores the potential of producing flavonoids through solid-state fermentation (SSF) using agro-industrial residues as substrates for filamentous fungi. *Trichoderma harzianum*, *Trichoderma viride* and *Beauveria bassiana* were evaluated for their ability to increase total flavonoids content on different substrates, in a screening process at laboratory scale, achieving a production of 0.53 ± 0.09 mg of total flavonoids per gram of dry matter (mg g^−1^dm) with *T. harzianum* in a blend of 30% spent coffee grounds (SCG) and 70% brewers spent grain (BSG) as carbon source. Subsequently, the bioprocess was optimized on 0.5 L packed bed bioreactors regarding cultivation time and productivity. A design of experiments was conducted to evaluate the influence of substrate composition and moisture content on the production of the desired metabolites, as well as the effect of temperature variation, resulting in an increase in total flavonoid production to 0.75 ± 0.14 mg g^−1^dm. Finally, the process was scaled-up under optimized conditions in a 22 L packed bed bioreactor, reaching a production of 0.43 ± 0.07 mg g^−1^dm. These results demonstrate the feasibility of valorizing agro-industrial residues through SSF using biopesticidal fungi, providing a scalable bioprocess to produce flavonoid-rich bioinputs.

**Graphical abstract:**

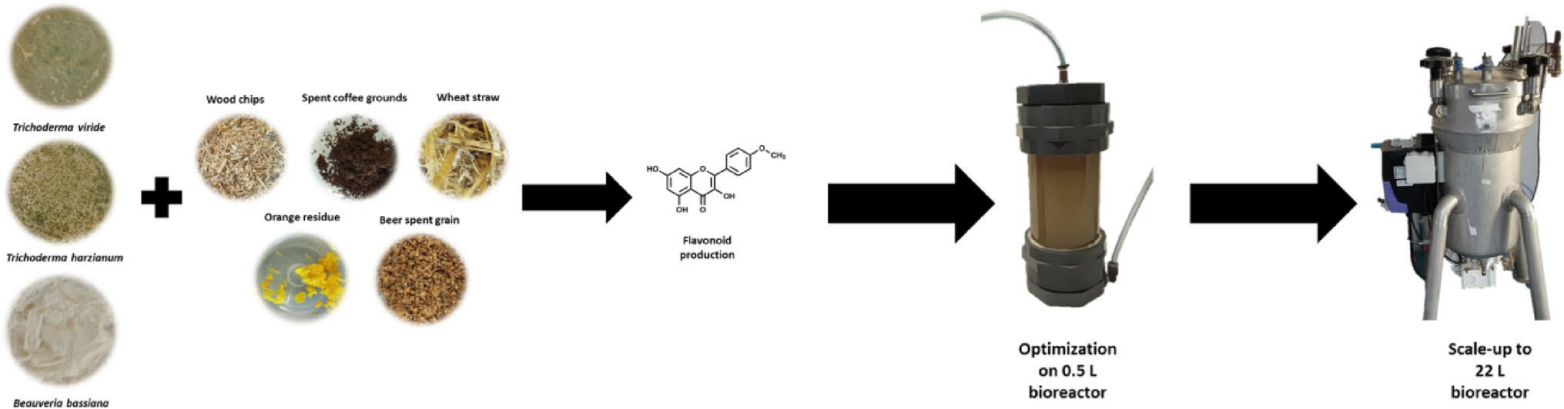

**Supplementary Information:**

The online version contains supplementary material available at 10.1007/s00449-026-03316-8.

## Introduction

The bioinputs market in agriculture, particularly in biological control, has grown significantly over the past decade due to reduced agrochemical reliance, low-carbon solutions, biodiversity preservation, and global climate agreements like COP26 [1]. Valued at $15 billion in 2023, the bioinputs market, including control products, inoculants, biostimulants and solubilizers, is projected to grow annually by 13% to 14%, reaching approximately $45 billion by 2032 [2]. However, the rapid expansion of this market al.so imposes significant pressure on production systems, demanding scalable, cost-efficient and resource-optimized manufacturing strategies capable of meeting industrial volumes without compromising sustainability goals.

Among bioinputs to control, *Beauveria bassiana* is widely used due to its insecticidal activity, mainly against *Bemisia tabaci* race B (whitefly), *Dalbulus maidis* (corn leafhopper) and *Hypothenemus hampei* (coffee berry borer), also being effective on another 700 arthropod species. Its adoption is driven by high efficacy and low risk to humans and to the environment [3, 4]. This insecticidal action occurs through the infection and colonization of the host, which is facilitated by the production of hydrolytic enzymes and toxic metabolites. Fungi from *Trichoderma* genus are also extensively used and researched for their ability to combat phytopathogenic fungi and nematodes through mechanisms such as mycoparasitism, competition, antibiosis, and induction of resistances [5]. *Beauveria bassiana* and different strains of *Trichoderma harzianum* and *T. reesei* produce a wide range of bioactive compounds, including alkaloids, flavonoids, phenolics, lactones, quinones, triterpenes, and sterols, which exhibit antagonistic activity against a wide range of insects and microbial pathogens [6, 7].

Within these compounds, flavonoids emerge as highly promising for agricultural applications. Beyond their antimicrobial properties, flavonoids also mitigate abiotic stresses, including excessive ultraviolet (UV) radiation and water deficiency. Moreover, they play a crucial role as signalling agents between plants, fostering the colonization of soil microbiota. This symbiotic relationship, in turn, enhances nutrient availability in soil, contributing to plant health and growth [8]. Despite their well-documented biological functions, flavonoids remain underexploited as target metabolites in fungal-based bioinputs, particularly at industrial scale. Their combined antimicrobial and plant-stimulatory properties suggest a potential dual-function product, reducing the need for multiple agricultural inputs.

Industrial production of fungal bioinputs is commonly performed using solid-state fermentation (SSF), a biotechnological process where microorganisms develop on a moist solid substrate that serves both as a carbon source and physical support, without free-flowing water. The porous structure of the substrate facilitates aeration through air-filled interparticle spaces, while moisture retained within the solid matrix ensures favourable conditions for microbial growth [9]. SSF allows the use of agro-industrial residues as substrates in a system that resembles natural environments, reducing costs and contamination risks and increases spores and metabolite concentrations [10–12]. This type of bioprocess becomes even more relevant when the volume of organic waste generated is considered.

In 2023, around 32% of global food production was not effectively utilized, with 12% corresponding to food loss and 20% to food waste [13]. There is a wide range of residues that can be used as substrates in SSF, like brewers spent grain (BSG), whose annual generation is estimated at 39 million tons [14, 15]. Similarly, 15 million tons of spent coffee grounds (SCG), constituting 45% of coffee byproducts, arise from beverage preparation [16]. Sweet orange production generates around 22 million tons of residue yearly, as only 45–60% of the fruit is used for juice [17]. Wheat straw, a major byproduct of cereal consumption, and wood chips, a residue from forest management, also hold great promise for SSF applications [18].

Each of these materials offers unique biochemical properties. BSG contains carbon, proteins, phenolic compounds, and antioxidants, making it ideal for microorganism growth and secondary metabolism stimulation [19]. SCG, rich in nutrients, enhances soil fertility by improving its chemical and physical properties [20]. Orange residues are nutrient-dense, providing vitamins, minerals, and phytochemicals like flavonoids and carotenoids, with applications ranging from nutrition to bioprocessing [21]. These properties underline their potential for use in sustainable biotechnological innovations. From an engineering standpoint, substrate selection directly influences mass transfer, aeration, moisture retention and metabolic yields in SSF systems. Therefore, evaluating structurally and chemically diverse residues becomes critical for process optimization and scale-up feasibility.

Wood chips, often treated as waste or burned for energy, present an underused resource for SSF. Not only their lignocellulosic composition makes them suitable for bioconversion into valuable products, offering a sustainable solution to environmental pollution, but they also can be used as bulking agent or support material. Integrating these substrates into SSF not only valorises waste but also contributes to circular economy, reducing the environmental burden of agro-industrial residues [22, 23].

However, SSF industrial processes are poorly automated due to the lack of engineering studies, whereas the most used matrices are also used as human feed, such as rice and maize [24]. The escalating global adoption of agricultural bioinputs underscores the urgency for fresh research endeavours aimed at refining technologies to render production processes more cost-effective and sustainable. Additionally, there is a growing emphasis on enhancing the multifunctionality of portfolios by gaining deeper insights into the untapped potential of commercial microorganisms. Consequently, the current proposal seeks to assess the efficacy of different carbon sources derived from agro-industrial wastes, worldwide generated, to produce flavonoids by SSF of filamentous fungi, which are already commercially employed as biodefensives.

Based on the potential of certain filamentous fungi to synthesize flavonoids and the advantages of solid-state fermentation (SSF) for agro-industrial waste valorisation, we hypothesize that these residues can serve as effective substrates for producing flavonoid-rich bioinputs with agricultural relevance. This study aims to identify suitable fungal–substrate combinations, optimize the SSF process parameters, and assess the scalability of the bioprocess in packed-bed bioreactors. To our knowledge, this is the first work on flavonoid production by SSF using *Trichoderma* spp. scaled up to a 22 L reactor, providing a practical framework for future applications in sustainable agriculture.

## Materials and methods

### Preservation and maintenance of microorganisms

This study used three fungal strains: *Beauveria bassiana* (CECT, 20374, isolated from *Dociostaurus maroccanus* before December 22, 1999*)*; *Trichoderma harzianum* (CECT 2929, isolated from soil in the United Kingdom before January 23, 1991); and *Trichoderma viride* (CECT 2942, isolated from soil in the United Kingdom before January 23, 1991). They were kept in cryogenic tubes with a sterile 20% (v/v) glycerol solution at a temperature of -80 °C [25], and subsequently *B. bassiana* were cultured on potato dextrose agar, while both *Trichoderma* strains in malt extract agar (MEA). Both strains were cultured for 240 h at 25 °C. Both *Trichoderma* strains followed a 12 h–12 h light-dark regime.

For the inoculum, a spore suspension was used, obtained from superficial scraping of Petri dishes or inclined Erlenmeyer flasks. To standardize inoculum concentration at 10^7^ spores/mL, counting was carried out in an improved Neubauer chamber, with the aid of an optical microscope as described by Prado et al., 2019 [26].

### Raw materials used in the composition of the cultivation medium

To compose the solid porous matrix, four different residues were evaluated as carbon and nitrogen sources. To increase the porosity of the solid media, wood chips were used. The solid porous media were the substrate itself, serving as carbon source for the growth of fungi and the consequent production of the metabolites of interest.

Orange residue (OR, from orange juice machine at the bar of Escola d’Enginyeria at UAB); spent coffee grounds (SCG, from bar of Escola d’Enginyeria at UAB); brewer’s spent grain (BSG, from Cervesa del Montseny S.L., Sant Miquel de Balenyà); wheat straw (WS, from Universitat Autònoma de Barcelona (UAB) experimental farms); and wood chips (WC, from Acalora, Ivars d’Urgell) were tested. Upon collection, all substrates were partitioned into plastic bags and stored with no additional pretreatment until use: OR, SCG and BSG were stored into cold chamber due to their higher moisture content (> 50%), whereas WC and WS were stored at room temperature due to having moisture content lower than 10%. This approach ensured minimal degradation prior to fermentation while maintaining the substrates in conditions comparable to industrial practice. The main characteristics of these materials are summarized in Supplementary information, including moisture content, dry matter, organic matter, pH, bulk density, air-filled porosity (AFP_R_), total flavonoids, water holding capacity (WHC), and total sugar content.

### Flask-scale solid state fermentation (SSF)

Flask-scale tests were carried out to evaluate the feasibility of microbial growth and sporulation on each of the previously mentioned residues, as well as to assess their potential for flavonoid production. All fermentations were conducted in triplicate using 500 mL Erlenmeyer flasks with cotton roll closures to allow gas exchange and prevent contamination. Its composition was 91.5% (w/w) of each individual carbon source and 8.5% (w/w) of wood chips. When working with WS, 5 g of dry substrate were used per flask without wood chips addition, due to the volume occupied by WS and its high porosity [27]. When working with this substrate, moisture content was adjusted to a minimum value of 50% by adding distilled water in wet wight %, based on the required volume to reach the target % moisture. After adjustment, the flasks were sterilized (121 °C, 1.1 atm, 20 min), cooled in a laminar flow chamber, and inoculated with 200 µL of standardized spore suspension of 10^7^ spores/mL. Fungal cultivation lasted 120 h at 25 °C in a static incubator [28].

### Evaluation of substrate composition on 0.5 L bioreactor

Aiming to evaluate a better substrate and porosity approach for flavonoid production, a substrate blending test was conducted using *Trichoderma harzianum* in a 0.5 L (total volume) packed-bed bioreactor. The bioreactors were cylindrical, PVC-made and completely closed except for air inlet (bottom) and outlet (top). The bioreactors were maintained at constant temperature using a heated water bath without mechanical agitation. As they could not be autoclaved, they were chemically sterilized by immersion in bleach for 24 h before use. Inoculation and packed-bed assembly were performed in a laminar flow chamber. An airflow meter ensured constant airflow rate, with air humidified before entering from the bottom. Exhaust air passed through a water trap to capture excess moisture before reaching an oxygen sensor for monitoring [29]. Specific oxygen uptake rate (sOUR) was calculated according to Eq. 1.

1 $$\begin{gathered} sOUR = F \times \left( {0,{\mathrm{2}}0{\text{9 }} - yO_{2} } \right) \times \hfill \\ \,\,\,\,\,\,\,\,\,\,\,\,\,\,\,(P \times {\mathrm{32}} \times {\mathrm{6}}0 \times {\mathrm{1}}000^{{\mathrm{a}}} )/(R \times T \times DW \times {\mathrm{1}}000^{{\mathrm{b}}} ) \hfill \\ \end{gathered}$$

where sOUR is the specific Oxygen Uptake Rate (g O_2_ kg^− 1^ DM h^− 1^); F is airflow rate (mL min^− 1^); yO_2_ is the oxygen molar fraction in the exhaust gas strain (mol O_2_ mol^− 1^); P is the pressure of the system, assumed constant at 101,325 Pa (atmospheric); 32 is the oxygen molecular weight (g O_2_ mol^− 1^ O_2_); 60 is the conversion factor from minute to hour; 1000^a^ is the conversion factor of mL to L; R is ideal gas constant (8310 Pa L K^− 1^ mol^− 1^); T is the temperature at which F is measured (K); DW is the initial dry weight of solids in the reactor (g); and 1000^b^ is the conversion factor of g to kg.

The fermentation was executed in duplicate at 25 °C during 120 h, with an aeration rate of 100 mL min^− 1^. Flavonoid analysis was carried out as described in 2.7. In this test, 35 g of dry substrate were used, of which 30% corresponded to WC as an inert material and 70% to a mixture of BSG and SCG. For the proportion of this last mixture, the air-filled porosity of the materials and their humidity were considered.

After determining the viability of the mixture of the best carbon sources and substrate composition, a time course study was conducted for *T. viride* and *T. harzianum* to evaluate total flavonoid production over 168 h. Samples were collected every 24 h, sacrificing one bioreactor per time point, with an aeration rate of 50 mL min^− 1^. The flow rate adjustment in this experiment was based both on observations from the previous experiment, in which higher superficial velocities were found to be unnecessary for the proper development of the process, and on the findings reported by Sala et al. (2019) [18]. Based on this preliminary data, the flow rate was reduced by 50% to optimize operating conditions, minimize energy consumption, and maintain system efficiency.

### Optimization of the flavonoid production process

After selecting the optimal fungal strain, carbon source combination, and fermentation time for flavonoid production, a study was conducted to optimize process conditions by evaluating two independent variables: substrate mixture and moisture content. The quantity of wood chips accounted for 50% of the total weight, while the remaining 50% consisted of varying proportions of brewers’ spent grain (BSG) and spent coffee grounds (SCG). A factorial design was proposed and analysed using Design Expert 12 ^®^ software, including 11 experiments with 2 factors, being different combinations of BSG levels (ranging from − 1 to 1, corresponding to 30% to 70% of the BSG: SCG mixture) and moisture levels (from − 1 to 1, corresponding to 20% to 60%), as shown on Table 1. The response variable measured was total flavonoid production.

**Table 1:** Matrix of experiments according to factorial design

Experiment	BSG (%)		Moisture (%)	
	Coded	Uncoded	Coded	Uncoded
1	0	50	0	40
2	1	70	-1	20
3	1	70	1	60
4	1	70	-1	20
5	1	70	1	60
6	-1	30	-1	20
7	0	50	0	40
8	-1	30	1	60
9	-1	30	-1	20
10	-1	30	1	60
11	0	50	0	40

After determining the best substrate conditions, an experiment to evaluate the effect of temperature on total flavonoids production was performed. Using the best fungal strain, three temperatures (25, 30 and 35 °C) were evaluated throughout four days of cultivation.

### Scale-up to 22 L packed-bed bioreactor

Based on the optimal conditions obtained in the experimental design, a scale-up test was carried in a 22 L packed-bed bioreactor. The 22 L bioreactor consisted in a removable basket (48 cm height, 24.5 cm diameter) with a working volume of 90% of its capacity. Oxygen monitoring system was the same as in the 0.5 L bioreactor system [30]. Airflow was set at 3000 mL min^− 1^ and the bulking material-to-substrate ratio was adjusted to 60:40 (wet basis) to maintain AFP_R_ around 70% and prevent compaction, resulting in 7500 g of inoculated substrate [31]. Temperature was monitored at multiple bed heights within the reactor, specifically at 0 cm (base of the reactor), 12 cm, 24 cm, and 36 cm using temperature sensors (T button sensors, iButton, Thermochron, United Kingdom), Sensotrans). The bioreactor was sanitized with water, bleach and alcohol before and after use, while the basket was sterilized (121 °C, 1.1 atm, 20 min). Inoculation was performed in a flow chamber. Samples were collected on days 3 and 4.

### Extraction and quantification of flavonoid production by colorimetric method

At the end of each cultivation, solid-liquid extraction was performed with acidified methanol (85% of 70% methanol solution and 15% of 10% acetic acid solution, all in % v/v) in an ultrasonic bath. Then, the solid fraction was separated by filtration through synthetic fabric. Finally, the quantification of total flavonoids in the supernatant of the crude extract was analysed in which 1 mL of extract reacts with 250 µL of aluminum chloride (5%) in a dark environment for 30 min and cold centrifuged (10000 rpm, 4 °C for 10 min) for subsequent reading on a UV-Vis spectrophotometer at 425 nm [24].

### Characterization of raw materials in the composition of the cultivation medium

Regular parameters were determined according to the standard procedures included in “Test Methods for the Examination of Composting and Compost” [32].

#### Bulk density

Bulk density (BD) is defined as a sample’s weight per volume unity. BD was calculated in a wet basis and used for porosity calculations, dividing the sample weight by the sample volume.

#### Moisture content and dry matter

Moisture content (MC) and dry matter (DM) determination were performed by gravimetric analysis. Samples (5 to 10 g) were placed in a previously weighted crucible and left in an air oven at 105 °C for at least 24 h to ensure the dryness of the sample. The crucible with the dried sample was weighed after cooling, and MC and DM were calculated.

#### pH

pH was determined utilizing the aqueous extract obtained after mixing the sample with distilled water in a 1:5 w/v ratio. The sample was shaken at room temperature for 30 min to solubilize the salts into the supernatant. pH was measured with an electrometric pH meter (Crison^®^, micropH2001).

#### Air-filled porosity (AFP_R_)

Air-filled porosity (AFP_R_) is defined as the volume fraction of air (usually reported in a percentage basis) in a porous matrix. It was calculated according to Eq. 2 [33].

2 $$\begin{gathered} AFP_{R} = {\text{ 1}} - BD_{t} \times \left\{ {\left[ {({\mathrm{1}} - - DM)/\left( {D_{W} } \right)} \right]} \right. \hfill \\ + \left. {\left. {(DM \times OM){\text{ }}/PD_{{OM}} } \right] + DM \times ({\mathrm{1}} - - OM))/{\text{ PD}}_{{{\mathrm{ash}}}} ]} \right\} \hfill \\ \end{gathered}$$

where: AFP_R_: air-filled porosity (%); BD_t_: total bulk density on a wet basis (kg m^− 3^); DM: dry matter on a wet basis (%); OM: organic matter on a dry basis (%); D_w_: water density (1000 kg m^− 3^); PD_OM_: organic fraction particle density (1600 kg m^− 3^); PD_ash_: ash particle density (2500 kg m^− 3^).

#### Total sugar content

Total sugar content (TSC) was determined using the anthrone method during the initial characterization of the substrates to estimate total carbohydrate content and assess their suitability for fermentation [34]. Sugars were extracted from dry samples by mixing them with distilled water (1:10 w/v), incubating at 50 °C for 15 min, and centrifuging at 4000 rpm for 10 min. The extraction was repeated twice, and the supernatant was filtered (0.45 μm). Anthrone reagent was freshly prepared with 200 mg anthrone in 100 mL cold 95% sulfuric acid. A reaction mixture of 1 mL sample supernatant and 4 mL anthrone reagent was heated in a boiling water bath for 8 min, then rapidly cooled. Absorbance was measured at 630 nm using a spectrophotometer (Varian Cary50 Bio, Agilent Technologies). Distilled water was used as blank instead of 1 mL sample supernatant. A glucose calibration curve (R² = 0.9907) was used for quantification at 6 different concentrations ranging from 0 to 0.1 mg/mL. Total sugar content was expressed as gram of glucose equivalent per gram of dry matter according to Eq. 3.

3 $$TSC\,=\,C/P$$

where: TSC: total sugar content (g /gdm); C: concentration of glucose equivalents (g/ L); P: weight of the dry sample (g); V: total volume of the supernatant (L).

#### Water holding capacity (WHC)

Water holding capacity (WHC) is defined as the capacity of a material to retain water. Initially, the material was washed with distilled water and dried at 65 °C for 24 h. Then, a known mass of material was transferred to a cylindrical tube with a net on the bottom and was saturated with water. The material was allowed to drain for 1 h. The resulting sample weight gained due to retained moisture represents the relative WHC of the material.

### Colony-forming unit (CFU) analysis

Colony-forming unit (CFU) analysis was carried out following the method described by [35]. Briefly, 10 g of fermented substrate was serially diluted in 90 mL of a 0.1% (v/v) Tween 80 solution, shaken for 20 min at 150 rpm and subsequently plated onto a standard agar medium. The plates were incubated at 25 °C for 48 h. After incubation, colonies were counted, and the total viable cell count was determined by correcting for the respective dilution factors.

### C/N ratio

C/N analysis was performed only for the three most promising substrates by means of chemical elemental (C, H, N and S) analysis by Servei d’Anàlisi Química (SAQ) in UAB, for initial characterization purposes. The analysis was carried out using a CHNS elemental analyzer Flash 2000 (Thermo Scientific). Samples were combusted at 1200 °C with air excess and quantification was performed by means of gas chromatography. C/N ratio analyses corresponding to the rest of substrates used can be found in other works of the same authors [31, 36].

### statistical analysis

All flask-scale fermentations were performed in biological triplicate. From each flask, samples were collected and subsequently analysed in technical triplicate for all analytical determinations, including substrate characterization, colony-forming unit (CFU) quantification, and flavonoid production. Packed-bed bioreactor fermentations (0.5 L) were conducted in duplicate, with samples taken from both units, and all corresponding analyses were performed in technical triplicate. For the 22-L packed-bed bioreactor, samples were collected from three vertical positions (upper, middle, and lower sections), with three technical samples obtained from each region; with a total of 9 samples in the whole reactor volume. The resulting data were subjected to analysis of variance (ANOVA) and to the Shapiro–Wilk normality test, and, as they followed a normal distribution, statistically significant differences were identified using Tukey’s test (*p* ≤ 0.05).

## Results and discussion

### Flask-scale solid state fermentation (SSF)

A screening was conducted to evaluate the suitability of different fungal strains and agro-industrial residues for bioconversion processes. *B. bassiana*, *T. harzianum*, and *T. viride* were cultivated in flask-scale tests using four different substrates: OR, SCG, BSG and WS. The evaluation focused on their capacity to support fungal growth, sporulation, and flavonoid production, highlighting the most promising strain–substrate combinations for further development. Table 2 shows results obtained after fermentation of the three strains for each raw material tested.

**Table 2:** Flavonoid production and CFU content after SSF performed with the three strains

Substrate/ Strain	Trichoderma viride				Trichoderma harzianum			Beauveria bassiana					
	Total Production (mg g^−1^dm)	Yield (%)	CFU g^−1^dm		Total Production (mg g^−1^dm)	Yield (%)	CFU g^−1^dm	Total Production (mg g^−1^dm)			Yield (%)	CFU g^−1^dm	
**Wheat straw**	0.16 ± 0.08^cd^	29.37	8.75E + 09		-0.22 ± 0.03^e^	–36.13	2.81E + 09		–	–		–	
**Orange residue**	0.06 ± 0.05^d^	68.05	5.50E + 08		-0.12 ± 0.05^e^	–11.64	9.04E + 08		–	–		–	
**Spent coffee grounds**	0.71 ± 0.21^a^	57.97	2.74E + 08		0.68 ± 0.10^a^	60.24	1.40E + 08		–	–		–	
**Brewer’s spent grain**	0.31 ± 0.02^bc^	300.06	3.51E + 09		0.35 ± 0.02^b^	379.39	5.26E + 10		0.12 ± 0.01^cd^	97.19		7.30E + 08	

*gdm = gram of dry matter

Different letters indicate significant differences between the evaluated groups (*p* < 0.05) based on the Tukey analysis

*Trichoderma viride* grew on all four tested substrates, resulting in 8.8 × 10^9^ CFU g^−1^dm for WS, 5.5 × 10^8^ CFU g^−1^dm for OR, 2.7 × 10^8^ CFU g^−1^dm for SCG, and 3.7 × 10^9^ CFU g^−1^dm for BSG. Growth was more homogeneous on the BSG and OR substrates, concentrated on the surface in SCG, and more dispersed in WS.

These values are promising when compared to most commercial biopesticides that have *T. viride* as the main active compound. Wettable powder (WP) formulations typically contain between 2.0 × 10⁶ CFU g⁻¹ and 1.0 × 10⁹ CFU g⁻¹ [37]. In this study, the values obtained (3.6 × 10⁷ CFU g⁻¹ to 1.0 × 10¹⁰ CFU g⁻¹) indicate strong potential for real-world applications, especially considering that no downstream processing or formulation optimization was performed.

Physicochemical properties, such as moisture content, organic matter, and AFPR, remained relatively stable, likely due to the small substrate quantity and the uniform aeration in Erlenmeyer flasks. The thin layer of substrate in these flasks likely facilitated even aeration across the entire surface, contributing to the stability of these parameters [38]. Beyond their stability during the fermentation period, these properties are highly relevant for future applications of the substrates. Moisture content and WHC directly influence microbial activity and oxygen diffusion, playing a critical role in solid-state fermentation performance. Organic matter content reflects the availability of biodegradable material, while bulk density affects aeration efficiency and heat transfer within larger-scale systems. Additionally, total sugar content and flavonoid levels help determine the potential of each substrate as a nutrient source or as a source of bioactive compounds. These characteristics collectively support informed substrate selection for future studies and industrial applications, especially when scaling up beyond flask-level experiments.

Moisture content and WHC directly influence microbial activity and oxygen diffusion, playing a critical role in solid-state fermentation performance. Water holding capacity relates to maximum amount of water that can be hold in the substrate without leaching. As such, substrate with high WHS can potentially reach higher moisture values, which might be preferrable for SSF, leading to higher growth of the fungus.

A major difference was only observed with pH, in which WS varied from 5.8 to 7.0, OR from 4.2 to 3.7, SCG from 4.6 to 5.3 and BSG from 5.3 to 5.8. For 3 of the substrates used there was an increase in pH, but there was a reduction in OR, making it even more acidic. Similar behaviour was observed by Sala et al. 2021 [36], who reported a decrease in pH when using a comparable residue, orange peel. It is also known that such acidic conditions can negatively affect fungal growth and sporulation, as optimal development generally occurs at pH values between 5 and 7. This pH reduction in OR is likely linked to the natural release of organic acids during the hydrolysis of pectin-rich components, combined with the production of acidifying metabolites by the fungus when metabolizing its high soluble-sugar content. As citrus residues have limited buffering capacity, these mechanisms collectively drive a more pronounced pH decrease than in the other substrates.

Flavonoid content increased significantly in WS, SCG, and BSG, with BSG showing the highest yield with a 300.1% increase, followed by SCG at 58.0%, and WS at 29.4%. However, no statistically significant difference was noted for the OR. Table 2 shows flavonoid production, yield, and CFU production of all three microorganisms for each substrate.

Total flavonoid production was 0.3 mg g^−1^dm for BSG, 0.7 mg g^−1^dm for SCG and 0.2 mg g^−1^dm for WS. Thus, the substrates that stood out were BSG due to the highest yield (considering that the initial sample contained only 0.1 ± 0.0 mg g^−1^dm) and SCG (which resulted in the highest final quantity with a statistical difference of 1.9 ± 0.1 mg g^−1^dm).

*Trichoderma harzianum* also grew on all substrates, though with more heterogeneity, resulting in 1.4 × 10^9^CFU g^−1^dm for WS, 2.8 × 10^9^ CFU g^−1^dm for OR, 7.8 × 10^7^ CFU g^−1^dm for SCG and 2.73 × 10^10^ CFU g^−1^dm for BSG. Homogeneous growth occurred in BSG and WS, while OR and SCG showed spotty surface growth. Hence, homogeneous growth was obtained in the substrates presenting higher AFP_R_ values (WS and BSG), while results for lower AFP_R_ substrates (OR and SCG) were heterogeneous. These results, within the 1.0 × 10⁶ to 1.0 × 10¹⁰ CFU g^− 1^ range of commercial formulations, also indicate strong biotechnological potential.

In this case (Table 3), moisture and organic matter were mostly consistent across WS, SCG, and OR. However, BSG showed a moisture increase from 73.4% to 77.8% and OR displayed a notable AFP_R_ shift from 56.9% to 67.0%. pH varied across all substrates except for OR, which remained stable (3.9 to 3.6). Other substrates showed pH increases: WS (5.5 to 7.4), SCG (4.6 to 5.5), and BSG (5.1 to 6.6).

**Table 3:** Initial and final physicochemical and microbiological parameters for SSF fermentation with *T. viride* and *T. harzianum*. Values are the average of independent samples and its standard deviation

Microorganism	Parameter/Substrate	WS				OR + Wood Chips		SCG + WC		BSG + Wood Chips	
		Initial		Final		Initial	Final	Initial	Final	Initial	Final
***T. viride***	**Moisture content (%)**	55.0 ± 0.0		53.3 ± 2.7		72.8 ± 0.2	76.5 ± 0.3	53.6 ± 0.0	47.8 ± 0.5	71.3 ± 0.1	75.6 ± 0.4
	**pH**	5.8 ± 0.0		7.0 ± 0.0		4.2 ± 0.0	3.7 ± 0.1	4.6 ± 0.1	5.26 ± 0.1	5.3 ± 0.0	5.8 ± 0.1
	**Bulk Density (g/L)**	42.8		18.9		357.8	319.6	396.7	407.27	290.0	259.6
	**AFP**_**R**_ **(%)**	96.5		98.5		67.9	70.92	65.8	67.43	74.2	76.5
***T. harzianum***	**Moisture content (%)**	55.1 ± 0.0	54.4 ± 0.1		73.8 ± 0.4		73.74 ± 0.2	52.9 ± 0.9	51.49 ± 2.0	73.4 ± 0.0	77.8 ± 0.5
	**pH**	5.5 ± 0.0	7.4 ± 0.1		3.9 ± 0.0		3.65 ± 0.1	4.6 ± 0.0	5.5 ± 0.1	5.05 ± 0.0	6.6 ± 0.1
	**Bulk Density (g/L)**	40.8	32.8		478.7		366.7	401.3	402.9	316.2	281.3
	**AFP**_**R**_ **(%)**	96.6	97.3		56.9		67.02	67.0	67.1	71.6	74.3

*gdm = gram per dry matter

Final flavonoid contents were: WS (0.38 mg g^−1^dm), OR (0.90 mg g^−1^dm), SCG (1.80 mg g^−1^dm), and BSG (0.44 mg g^−1^dm). Both WS and OR showed flavonoids decrease, possibly due to nutrient consumption or compound biotransformation. Flavonoid production was highest in SCG (0.68 mg g^−1^dm, 60.2% yield) and BSG (0.35 mg g^−1^dm, 379.4% yield).

In contrast, *Beauveria bassiana* displayed limited growth, only thriving in BSG and WC media. In BSG, moisture rose from 73.4% to 75.6%, pH from 5.0 to 6.8, and porosity dropped from 75.4% to 73.0%. CFU g^−1^dm increased from 6.1 × 10⁷ to 7.3 × 10⁸, and flavonoids from 0.12 ± 0.01 to 0.24 ± 0.01 mg g^−1^dm (97.2% yield), as shown in Table 2, which presents a comparative analysis across all conditions. In the rest of the tested substrates, pH was too acidic (around 4.0 in OR) or sugar availability too low (< 7.5 in WS and SCG) for *B. bassiana* to thrive.

In WS, *T. viride* increased flavonoids, while *T. harzianum* caused a decrease. In OR, neither strain significantly increased total flavonoids concentration, and *T. harzianum* reduced them. Both strains enhanced flavonoid levels in SCG and BSG, with no statistical difference between them in SCG. However, *T. harzianum* outperformed *T. viride* in BSG. Although *B. bassiana* also increased flavonoid content in BSG, its production was lower than that of both *Trichoderma* strains.

The results of this preliminary test using BSG as substrate demonstrated the ability to increase flavonoid content by 3.1-fold when fermented with *Trichoderma viride* and by 3.5-fold when fermented with *Trichoderma harzianum*. These increases are greater than those reported by Zhao et al. (2023), who observed a 2.97-fold increase in flavonoid content during the fermentation of mulberry leaves using *Aspergillus cristatus* [39]. Results were also promising with SCG as substrate, obtaining the highest flavonoid production out of all substrates (around 0.7 mg g^−1^dm with both *Trichoderma* strains). In contrast, both OR and WS results were inferior, mostly due to the acidic pH (OR) or the low availability of fermentable sugars (WS), which deemed both substrates as unsuitable for flavonoid production with the tested strains.

### Evaluation of substrate composition for *T. viride* fermentation on 0.5 L packed-bed bioreactor

To optimize flavonoid production, a substrate mixture was developed based on the best-performing individual components. Given that SCG and BSG not only supported the highest conidia yields, with statistically significant increases from initial to final samples, but also demonstrated strong flavonoid production (particularly SCG), a blend of 30% SCG and 70% BSG was selected as the carbon source. This ratio was selected to ensure suitable AFPR values for further scale up, and further supported by their complementary elemental profiles: wood chips had contained 47.9 ± 1.8% carbon, 6.4 ± 0.2% hydrogen, and 0.4 ± 0.2% nitrogen with no detectable sulfur, while SCG presented 49.7 ± 0.1% carbon, 7.1 ± 0.2% hydrogen, and 2.0 ± 0.0% nitrogen; BSG, in turn, had shown 45.9 ± 0.1% carbon, 6.7 ± 0.1% hydrogen, 3.0 ± 0.4% nitrogen, and was the only substrate containing sulfur (0.2 ± 0.0%). These values confirmed that all residues were rich carbon sources with similar hydrogen levels, and that BSG uniquely contributed higher nitrogen and sulfur availability, improving the nutritional balance of the mixture. Consequently, the formulation, designed to balance high flavonoid synthesis with structural integrity (avoiding compaction seen in coffee grounds alone), consisted of 17.2 g BSG, 7.4 g SCG, and 11.3 g WC.

The mixture was fermented using *Trichoderma viride*, as both *Trichoderma* strains had shown similar performance in flask-scale flavonoid enhancement. The process yielded a 2.8-fold increase in total flavonoids, rising from 0.19 ± 0.01 mg g^−1^dm to 0.53 ± 0.09 mg g^−1^dm. This corresponded to a net production of 0.34 mg g^−1^dm and a 174.0% yield. From a process engineering perspective, the combination of SCG and BSG not only enhanced flavonoid yield but also improved bed porosity and gas transfer, which are critical parameters in packed-bed SSF systems. This structural stability is particularly relevant for scale-up, where substrate compaction and oxygen limitations frequently compromise productivity.

**Fig. 1:**
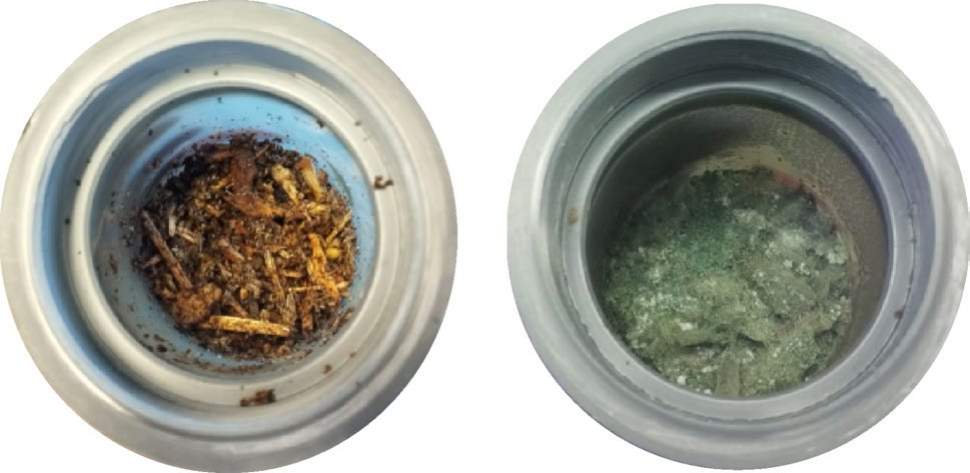
SSF with *T. viride* on 0.5 L bioreactors

Figure 1 illustrates the solid-state fermentation (SSF) process of *Trichoderma viride* in 0.5 L bioreactors. The image on the left depicts the initial substrate prior to fungal colonization, while the image on the right shows the substrate after incubation, evidencing extensive mycelial growth and marked visual changes.

**Fig. 2:**
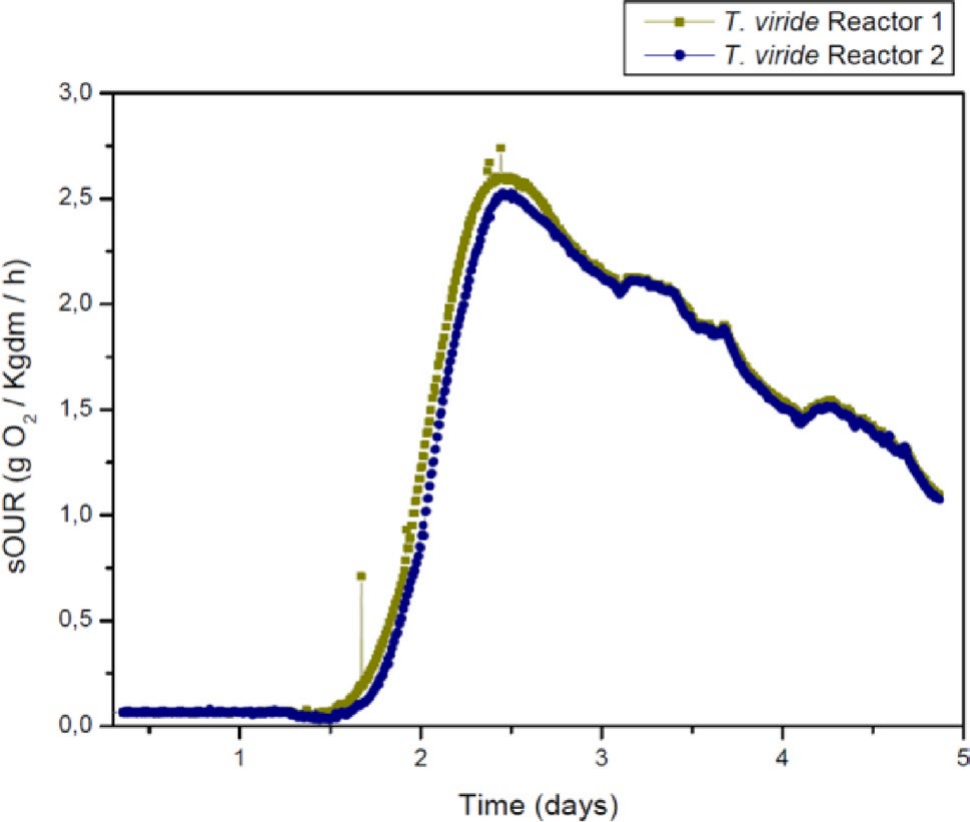
Respiratory profile of the duplicate costive of *T. viride* on 0.5 L bioreactors

Alongside this, robust fungal growth was observed, with CFUs reaching 1.7 × 10¹⁰ CFU g^−1^dm by the end of fermentation. The highest sOUR was recorded on day two, peaking at 2.5 ± 0.01 g O₂ kg^− 1^ DM h^− 1^ (Fig. 2), indicating active metabolism aligned with flavonoid synthesis.

Dulf et al. (2016) carried out fermentations using two types of plum by-products, including brandy distillery residues, and reported a 1.3-fold increase in total flavonoid content when *Rhizopus oligosporus* was employed, and a 1.2-fold increase with *Aspergillus niger*. In the case of plum pomace fermentation, both microorganisms yielded approximately a 1.4-fold increase in flavonoid content [40].

### Time course for *T. viride* and *T. harzianum* in 0.5 L packed-bed bioreactor

**Fig. 3:**
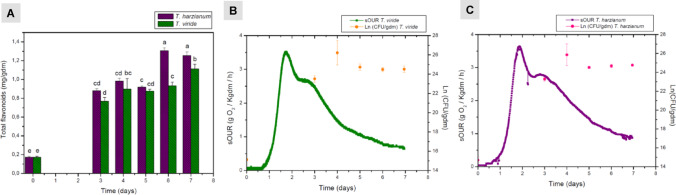
**a** Comparison of flavonoid content during time course between *T. viride* and *T. harzianum*. Different letters indicate significant differences between the evaluated groups (*p* < 0.05) based on the Tukey analysis. **b** Respiratory profile of *T. viride* in comparison with CFU/gdm during time course. **c** Respiratory profile of *T. harzianum* in comparison with CFU/gdm during time course

Figure 3a shows total flavonoid content obtained in the time course experiment. For *T. viride*, the highest quantity of flavonoids was reached after 7 days of cultivation, achieving 1.11 mg g^−1^dm, statistically different from the other quantities observed. This represents a production of 0.94 mg g^−1^dm. For *T. harzianum*, the highest quantity was obtained after 6 days of cultivation, being statistically equal to the quantity obtained after 7 days with 1.31 mg g^−1^dm, which represents a production of 1.14 mg g^−1^dm. Flavonoid productivity was also analysed during cultivation, in which both achieved the highest values between 72 and 96 h, *T. viride* with 8.30 ± 0.55 µg g^−1^dm h^− 1^ and *T. harzianum* with 9.84 ± 0.33 µg g^−1^dm h^− 1^. g O₂ Kg^− 1^ DM h^− 1^.

Figure 3b and c shows respiratory profile corresponding to both strains. Both microorganisms exhibited similar respiration profiles, with respiration peak occurring after approximately two days of fermentation, reaching 3.8 and 3.5 g O₂ Kg^− 1^ DM h^− 1^, for *T. harzianum* and *T. viride*, respectively. However, growth peak was observed on the fourth day of cultivation, signalling the end of the lag phase and the onset of the stationary phase. *T. harzianum* reached 1.7 × 10^11^ CFU g^−1^dm while *T. viride* reached 2.4 × 10^11^CFU g^−1^dm.

Both *T. viride* and *T. harzianum* demonstrated effective flavonoid production, with *T. harzianum* exhibiting superior yield and productivity. Given the similar respiratory profiles observed for both strains, characterized by peak oxygen uptake at 48 h and maximum microbial growth at 96 h, a fermentation period of 96 h was selected for subsequent stages of this study.

### Optimization of flavonoid production with *T. harzianum*

Table 4 shows the ANOVA obtained on the factorial design, proving that the model used in the factorial experimental design conducted for the microorganism *Trichoderma harzianum* was significant, with p-value of 0.0004. Looking into the variables, the percentage of brewers’ spent grain (BSG) in the mixture BSG: SCG was not statistically significant for flavonoid production. In contrast, moisture content and the interaction between moisture and BSG were significant factors.

**Table 4:** ANOVA results obtained in the Factorial Design

Source	Sum of squares	df	Mean square	F-value	*p*-value	
Model	0.6857	3	0.2286	33.52	0.0004	significant
A-BSG	0.0010	1	0.0010	0.1485	0.7133	
B-Moisture	0.6441	1	0.6441	94.45	< 0.00001	significant
AB	0.0406	1	0.0406	5.96	0.0504	

BSG: % in BSG: SCG mixture; Moisture: % w/w

**Fig. 4:**
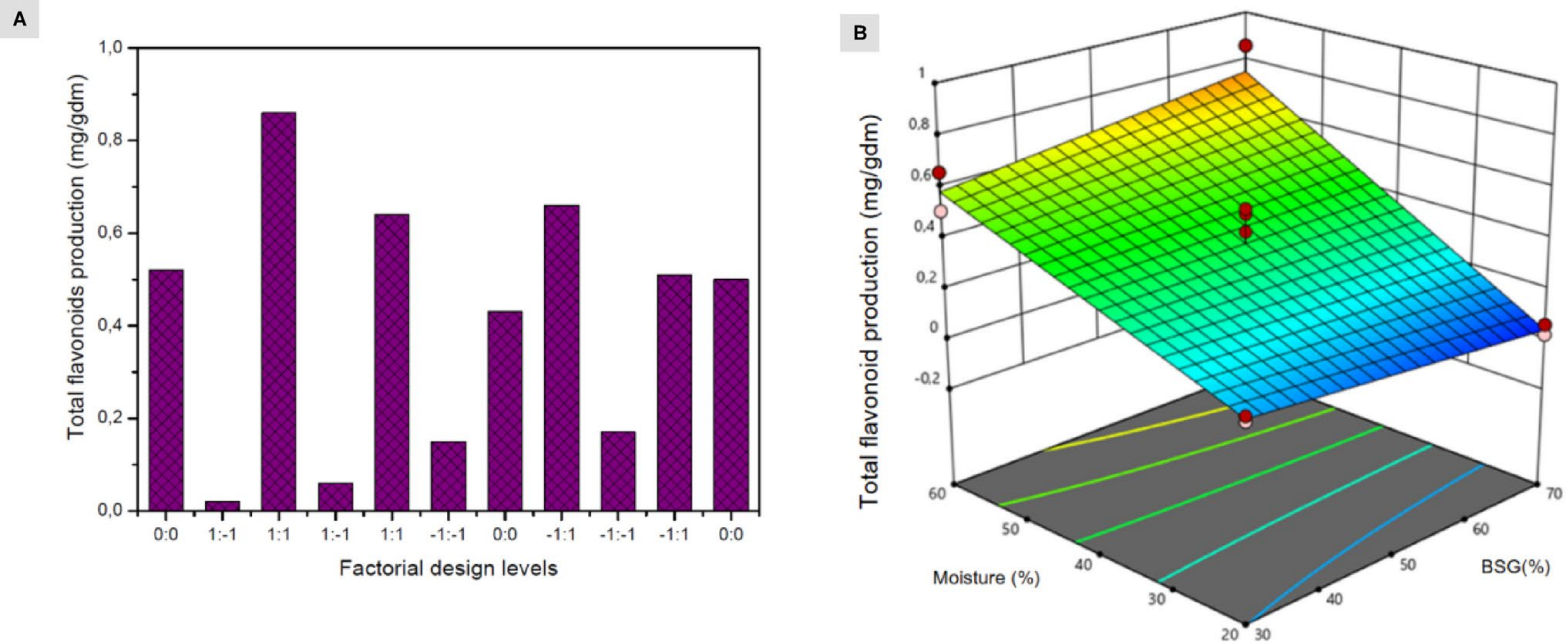
**a** Matrix of experiments according to factorial design. **b** Response surface of factorial design with *T. harzianum*

Figure 4a presents the results of total flavonoids for each level of the design. The highest production of total flavonoids for *T. harzianum*, amounting to 0.75 mg g^−1^dm, was achieved under conditions of 60% moisture and 70% BSG. While on experiments with 20% moisture, the production was between 0.02 and 0.17. Figure 4b shows the response surface obtained with the factorial design.

The respiratory profile showed similar behaviour in reactors where the duplicate conditions with 70% of BSG and 60% moisture led to the quickest respiration peak, which occurred after 36 h, reaching 2.5 g O₂ Kg^− 1^ DM h^− 1^. In contrast, reactors with low moisture content (20%) did not exhibit respiration activity, consequently, no visible microbial growth was observed. The respiration profiles of the central point, with 50% BSG and 40% moisture had peaks after two and a half days of fermentation reaching 1.5 g O₂ Kg^− 1^ DM h^− 1^, respectively. The profiles presented by 30% BSG and 60% moisture presented a peak of 2.7 g O₂ Kg^− 1^ DM h^− 1^ after 3 days.

In the colony-forming unit analyses, all reactors with low moisture levels showed values lower than the initial inoculum, indicating cell death over time. For the condition of 70% BSG and 20% moisture content, CFU g^−1^dm values started at 4.4 × 10^6^ and decreased to 1.9 × 10^5^ and 2.6 × 10^4^, respectively. With 30% of BSG and 20% moisture, the initial CFU g^−1^dm was 1.2 × 10^6^ declined to 6.7 × 10^4^ and 2.6 × 10^5^ by the end of cultivation. In contrast, all other conditions displayed similar growth levels between 1.3 × 10^10^ and 3.1 × 10^10^ CFU g^−1^dm.

The factorial design demonstrated that moisture content and its interaction with BSG significantly affected flavonoid production by *T. harzianum*, whereas BSG alone was not a determining factor. Maximum yield (0.75 mg g^−1^dm) was obtained at 60% moisture and 70% BSG. Respiratory and CFU analyses confirmed the necessity of adequate moisture content for microbial growth and flavonoid synthesis in solid-state fermentation.

### Effect of temperature on flavonoid production with *T. harzianum*

The effect of temperature on the microbial growth and flavonoid production was examined, considering that temperature increases are commonly observed during scale-up processes due to metabolic heat generation and reduced heat transfer efficiency [41]. Total flavonoid production was directly influenced by temperature variation. The highest flavonoid production, 0.41 ± 0.02 mg g^−1^dm, was achieved at 25 °C, followed by 0.29 ± 0.02 mg g^−1^dm at 30 °C, and 0.16 ± 0.05 mg g^−1^dm at 35 °C. Notably, the total amount of flavonoids reached 0.59 mg g^−1^dm at 25 °C.

**Fig. 5:**
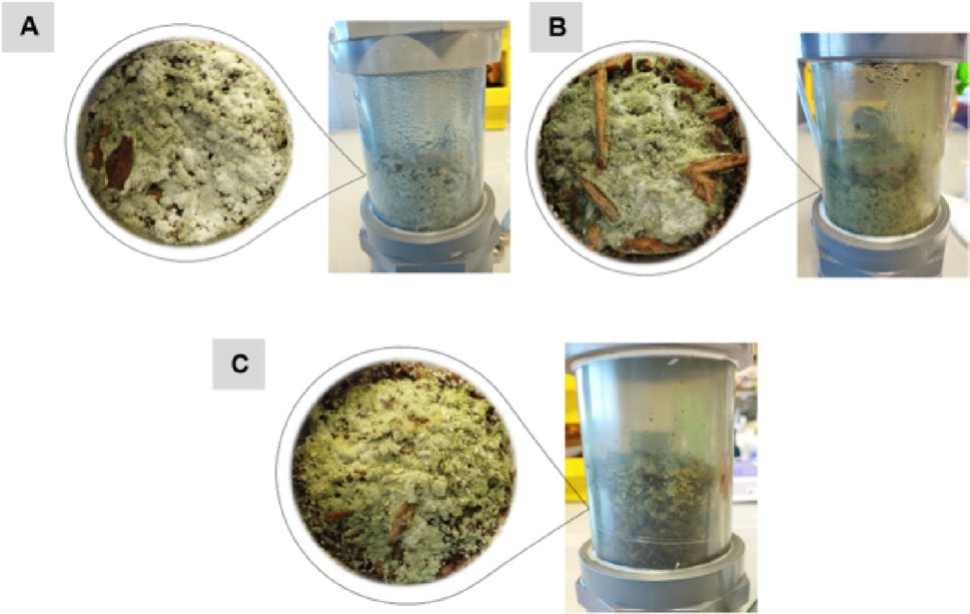
*T. harzianum*’s growth at **a** 25 °C **b** 30 °C **c** 35 °C

Microbial growth occurred homogeneously in the reactor at temperatures of 25 °C and 30 °C, as shown in Fig. 5. However, at a temperature of 35 °C, there was no growth in the middle region of the reactor, only in the lower and upper parts. This was likely due to the lower part being in direct contact with the air outlet, resulting in a milder temperature. On the other hand, the upper part was not submerged in the water bath, which may have also favoured growth. The CFU values at the end of fermentation were 3.3 × 10^10^ CFU g^−1^dm at 25 °C, 4.9 × 10^10^ CFU g^−1^dm at 30 °C, and 6.6 × 10^9^ CFU g^−1^dm at 35 °C.

The respiration profile showed that the culture at 30 °C reached the peak oxygen consumption more rapidly, after one day and a half of cultivation, with a rate of 2.9 ± 0.3 g O₂ Kg^− 1^ DM h^− 1^, aligning with expectations of accelerated microbial metabolism at this temperature. This was followed by the peak at 25 °C, which occurred after 44 h with an oxygen consumption rate of 2.6 ± 0.1 g O₂ Kg^− 1^ DM h^− 1^.

Thus, it was determined that the optimal temperature for flavonoid production is 25 °C. The *T. harzianum* strain grows better within the 25–30 °C range but can tolerate temperatures up to 35 °C, at which point growth inhibition begins. Growth accelerates at 30 °C but does not yield optimal flavonoid production.

### Scale-up on 22 L packed-bed bioreactor with optimized conditions

Solid-state fermentation (SSF) for flavonoid production remains poorly explored in the literature, particularly for agricultural applications, and existing studies are typically limited to small-scale experiments. In this context, the present study progresses from 0.5 L reactors to a 22 L packed-bed bioreactor, representing a relevant scale-up step for this system and contributing new insights at this intermediate scale.

**Fig. 6:**
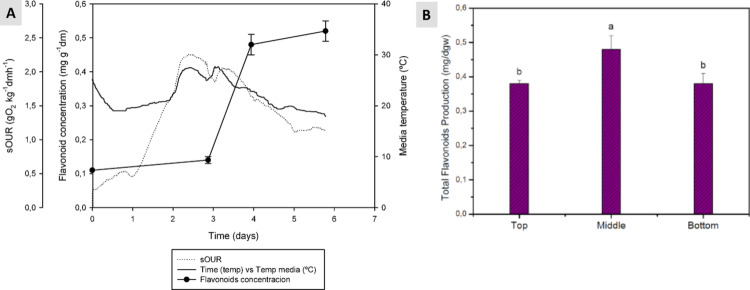
**(a.** Results obtained on the scale-up process: temperature variation, respiratory profile, flavonoid concentration **b.** Total flavonoid production quantified by reactor section

The bench-scale fermentation was successfully carried out in a 22 L packed-bed reactor. Initial moisture content was 54.7%, the AFP_R_ was 69.9%, and the inoculum was 4.2 × 10^6^ CFU g^−1^dm. During fermentation, the average temperature oscillated between 20 and 30 °C (Fig. 6a), with the highest value observed in the central positions of the reactor being 34 °C, tolerated by the used strain [31]. Growth was homogeneous throughout the reactor, reaching 1.7 × 10^10^ CFU g^−1^dm. Volume reduction was not observed, hence, no compaction occurred and AFP_R_ remained stable at 69.1% by the end of fermentation. Bulk density and air-filled porosity are critical parameters in fungal SSF scale-up. If not properly adjusted, the bed is prone to compress, and air does not flow properly throughout the solid matrix. This leads to areas in the reactor where the fungus might not grow or, even, anaerobic zones. If correctly adjusted, it contributes to a better heat transfer throughout the matrix. Incorrect assessment of these parameters may also lead to contamination. Consequently, their adjustment is critical when scaling to bench scale and beyond [31]. Temperature rise is also influenced by WHC and moisture. Even though a minimum temperature rise was not avoidable, WHC of the mixture used in this experiment was sufficient to allow working at moisture levels high enough to ensure the correct performance of the fermentation. When scaling, high amounts of metabolic heat are generated, hence, choosing a substrate with high WHC and with capacity to achieve a moisture value high enough is critical for the process. Hence, the mixture used in this work (60 − 40%, bulking material-substrate in wet basis) allowed working at a AFPR value around 70%, which is necessary to ensure proper air transfer in a 22 L packed-bed reactor, and at a WHC value sufficient to ensure the correct performance of the process.

The peak respiration rate occurred after two days, reaching 0.9 g O₂ Kg^− 1^ DM h^− 1^, as shown in Fig. 6a, which was lower due to the greater amount of bulking material and, consequently, a reduced amount of nutrients. The specific oxygen consumption related to the substrate at the peak respiration rate was 2.3 g O₂ Kg^− 1^ DS h^− 1^, (ds being dry substrate, excluding the bulking material).

Flavonoid production was 0.48 ± 0.04 mg g^−1^dm in the centre of the reactor and 0.38 ± 0.03 mg g^−1^dm in the upper and lower sections, representing a 3.9-fold increase to the initial quantity of flavonoids. From the samples collected on the third and fourth days of cultivation, the process productivity was calculated as 0.43 µg g-1 dm h-1 at 3 days, 3.82 µg g^−1^dm h^− 1^ at 4 days, and 1.89 µg g^−1^dm h^− 1^ at 6 days.

Given that 1996.5 gdm of substrate were used in the bioreactor for this batch and considering the average production at the end of cultivation was 0.43 ± 0.07 mg g^−1^dm, the total amount of flavonoids produced was 858.50 mg. The flavonoid yield obtained in the 22 L bioreactor (0.43 mg g⁻¹ dm) is within the same range reported for several plant matrices, many of which present values between 0.3 and 3 mg g⁻¹. Considering that microbial SSF is more reproducible, independent of plant cultivation, and capable of scaling without seasonal or agricultural constraints, the production achieved here can be regarded as significant. To our knowledge, no previous studies have demonstrated flavonoid production during SSF at liter-scale using kilogram-level substrate loads. Thus, the present work constitutes one of the first demonstrations of SSF-derived flavonoid production under bioreactor conditions, confirming that the obtained values are both technically relevant and meaningful from a bioprocessing and scalability perspective.

In terms of potential utilization, the flavonoids produced in this SSF system may be extracted through aqueous solid–liquid extraction, allowing simultaneous recovery of metabolites and viable spores for multifunctional agricultural formulations with both immediate (metabolite-driven) and long-term (spore-mediated) effects. When spore viability is not required, solvent-based extraction may be applied to maximize phenolic recovery, followed by solvent removal and incorporation of the concentrated extract into sprayable or wettable-powder products. Alternatively, the fermented solid itself could be processed into granular or pelletized formulations for direct soil application. These routes highlight the versatility and practical potential of the flavonoids generated at 22 L scale.

Table 5 presents a comparison between different modes of cultivation of *T. harzianum* strain through the optimization and scale-up process and its effect on growth and total flavonoid production. Growth of this strain remained consistent across all conditions. However, the production of total flavonoids was influenced by the amount of WC used as a bulking material. Higher quantities of WC resulted in reduced flavonoid production, likely due to WC being considered an inert material [21]. On the other hand, productivity was statistically equivalent in the 22 L bioreactor and the 0.5 L bioreactor in the cultivation containing 50% bulking material, being higher only in the cultivation with the largest amount of available substrate. Hence, successful scale-up of the flavonoid production process presented in this work up to a volume of 22 L was achieved. The promising results shown in this work pave the way for future scale-up of this process. However, more insights at this volume are mandatory to ensure a proper scale-up, as higher volumes will likely amplify heat generation and difficult mass transfer, the most important drawback present when scaling SSF and, particularly, the packed-bed configuration [42].

**Table 5:** Comparison between cultivations of *T. harzianum* in different packed-bed bioreactor scales

Bioreactor	Substrate Composition	AFP_*R*_ (%)	Moisture (%)	Airflow (mL min^− 1^)	sOUR (g O₂ Kg^− 1^ DM h^− 1^ )	sOUR (g O₂ Kg^− 1^ DS h^− 1^)	Total flavonoid production (mg g^−1^dm)	Yield (mg flavonoid g^−1^ds)	Productivity (mg L^− 1^)	CFU g^−1^DM	
0.5 L	30% WC, 49% BSG, 21% SCG (dry basis)	69.44	60.74	50	3.51	4.10	1.14	1.08	89.12 ± 3.20^a^		1.70 × 10^11^
0.5 L	50% WC, 35% BSG, 15% SCG (dry basis)	73.57	60.00	50	2.40	3.47	0.75	1.09	69.04 ± 2.10^b^		2.89 × 10^10^
22 L	60% WC, 28% BSG, 12% SCG (humid basis)	69.87	54.74	3000	0.90	2.25	0.43	0.69	63.57 ± 8.41^b^		1.66 × 10^10^

*gdm = gram of dry matter

*gds = gram of dry substrate

Different letters indicate significant differences between the evaluated groups (*p* < 0.05) based on the Tukey analysis

To assess spore yield and evaluate potential applications relative to commercial products, spore production and colony-forming unit (CFU) counts were compared in the final batch. This analysis is particularly relevant as these two metrics are currently used to determine the concentration of biopesticides and biostimulants as a final product. Values obtained were 3.92 × 10^10^ spores/gdm and 1.69 × 10^10^ CFU g^− 1^dm at the top of the reactor, 3.76 × 10^10^ spores/gdm and 1.69 × 10^10^ CFU g^− 1^dm at the middle of the reactor and at the bottom 4,36 × 10^10^ spores/gdm and 1.61 × 10^10^ CFU g^− 1^dm. Such results demonstrate their significant potential for field application, mainly when compared to Trichodermil^®^ from Koppert, which contains 46% of *Trichoderma harzianum* Rifai, strain ESALQ-1306, at a concentration of 2.0 × 10⁹ viable conidia/mL, or to NatuControl^®^ from Biotrop, which contains 1,15% p/p of *Trichoderma harzianum*, at a concentration of 1 × 10⁷ UFC g^− 1^ [43, 44]. Thus, in some points, the concentration reached up to 1000 times higher, which enables its use as an active ingredient in formulations, with the possibility of using additives that can improve stability, applicability, and durability of the final product. These results indicate that the process is not only technically scalable but also commercially relevant, as the obtained spore concentrations fall within or above the range required for marketable bioformulations. From an industrial standpoint, achieving both metabolite production and high spore density in a single SSF batch strengthens the feasibility of developing multifunctional bioinputs with reduced downstream processing requirements.

## Conclusions

Successful flavonoid production using *Trichoderma harzianum* was scaled up to a 22 L bench-scale packed-bed bioreactor, which represents a relevant step towards scaling SSF systems for agricultural applications. Although flavonoid production via SSF has been mainly studied at small scale, this study demonstrates the feasibility of intermediate-scale implementation using a 22 L packed-bed bioreactor. Previous reports on SSF for flavonoid production remain limited to small-scale experiments, generally involving only 2 to 15 g of dry substrate. In contrast, this study progressed from 0.5 L reactors (35.9 g dry matter) to a 22 L bioreactor (1.99 kg dry matter), offering a reproducible framework for intermediate-scale bioconversion processes.

Among the agro-industrial residues evaluated, BSG and SCG demonstrated the highest potential for supporting flavonoid production, with BSG being particularly favourable due to its higher porosity and superior support for fungal growth. Both *Trichoderma* species tested exhibited robust flavonoid yields and lower sensitivity to substrate type compared to *Beauveria bassiana*. A combination of BSG and SCG further enhanced fungal growth while sustaining substantial flavonoid synthesis. Flavonoid accumulation was observed to initiate around day six of cultivation for both *Trichoderma* strains, with peak productivity occurring between 72 and 96 h. *T. harzianum* consistently outperformed *T. viride* in flavonoid production, achieving the highest yields under optimized conditions of 60% moisture content, 70% BSG composition and an incubation temperature of 25 °C. Upon scale-up to bench-scale, *T. harzianum* maintained a total flavonoid yield of 0.43 mg g^−1^dm and produced 4.01 × 10¹⁰ spores/gdm, demonstrating both the efficiency and scalability of the process.

## Supplementary Information

Below is the link to the electronic supplementary material.

Supplementary Material 1

## Data Availability

Data will be made available on request.
