# Supplementary material for: Flavonoid production via solid-state fermentation of agro-industrial waste with biopesticidal fungi: screening, optimization and scale-up
Source: Bioprocess Biosyst Eng. 2026 Mar 23;49(5):1287–302. doi: 10.1007/s00449-026-03316-8 (PMC13263265; doi:10.1007/s00449-026-03316-8)
Supplement: Supplementary file 1 — Supplementary Material 1 [file 449_2026_3316_MOESM1_ESM.docx]

**Flavonoid production via solid-state fermentation of agro-industrial waste with biopesticidal fungi: Screening, optimization and scale-up.**

Fabíola Ribeiro de Oliveira^a^, Arnau Sala^b*^, Clarissa Okino-Delgado^a^, Teresa Gea^b^, Fernanda Perpétua Casciatori^a^.

^a^ Graduate Program of Chemical Engineering, Federal University of São Carlos (UFSCar), Rod. Washington Luiz km 235 SP 310, Bairro Monjolinho, 13565-905 São Carlos, SP, Brazil

^b^ Department of Chemical, Biological and Environmental Engineering, Universitat Autònoma de Barcelona, Edifici Q, Campus de Bellaterra, 08193 Cerdanyola del Vallès, Spain

* Corresponding author: Arnau Sala

ORCID: [0000-0001-7010-6984](https://www.scopus.com/redirect.uri?url=https://orcid.org/0000-0001-7010-6984&authorId=57205539961&origin=AuthorProfile&orcId=0000-0001-7010-6984&category=orcidLink)

Tel.: +34 669842411

E-mail address: Arnau.Sala@uab.cat

**Supplementary information**

**Table S1.** Characteristics of the raw substrates tested. Values are the average of independent samples and their standard deviation.

| **Substrate/Parameters** | **Wheat Straw** | **Wood Chips** | **Orange Residue** | **Brewer Spent Grain** | **Spent Coffee Grounds** |
| --- | --- | --- | --- | --- | --- |
| **Moisture content (%)** | 7.4 ± 0.8 | 6.4 ± 0.0 | 78.7 ± 0.5 | 76.1 ± 0.3 | 59.7 ± 0.3 |
| **Dry Matter (%)** | 92.6 ± 0.8 | 93.6 ± 0.0 | 21.3 ± 0.5 | 24.0 ± 0.3 | 40.3 ± 0.3 |
| **Organic matter (%)** | 97.0 ±1.7 | 94.5 ± 1.3 | 95.5 ± 1.3 | 88.8 ± 2.3 | 94.2 ± 1.7 |
| **pH** | 6.4 ± 0.0 | 4.8 ± 0.1 | 4.4 ± 0.0 | 5.7 ± 0.0 | 5.6 ± 0.0 |
| **Bulk Density (g/L)** | 21.0 ± 0.0 | 155.7 ± 15.3 | 496.0 ± 33.1 | 394.0 ± 38.7 | 469.6 ± 12.7 |
| **AFP_R_ (%)** | 98.6 | 90.1 | 54.5 | 64.4 | 60.4 |
| **Total flavonoids (mg g^-1^dm)** | 0.1 ± 0.0 | 0.1 ±0.0 | 1.0 ± 0.0 | 0.1 ± 0.0 | 1.3 ± 0.2 |
| **WHC (g_water_/gdm)** | 2.3 ± 0.8 | 2.0 ± 0.4 | 0.71 ± 0.3 | 2.6 ± 1.5 | 3.8 ± 0.5 |
| **Total Sugar Content (mg g^-1^dm)** | 7.4 ± 0.0 | 2.7 ± 0.0 | 111.4 ± 0.1 | 82.8 ± 0.1 | 6.1 ± 0.0 |

*gdm = gram of dry matter; AFP_R_ = air-filled porosity; WHC = water holding capacity
